# Development of repetitive transcranial magnetic stimulation for central poststroke pain: A review

**DOI:** 10.1097/MD.0000000000043441

**Published:** 2025-07-18

**Authors:** Nannan Yang, Tao Liu, Feiye Chen, Deheng Cui

**Affiliations:** a Department of Rehabilitation, The Second Hospital of Longyan, Longyan, Fujian Province, China; b Department of Urology, The Second Hospital of Longyan, Longyan, Fujian Province, China.

**Keywords:** central poststroke pain, mechanism, repetitive transcranial magnetic stimulation

## Abstract

The higher incidence of central poststroke pain (CPSP) has a severe negative impact on patients’ mood, sleep, recovery, and quality of life, with bad medication outcomes. On the contrary, noninvasive transcranial magnetic stimulation is safe, and its analgesic effect has been verified in clinical practice. Repetitive transcranial magnetic stimulation may treat CPSP by various mechanisms, including modulating immune responses, promoting neurogenesis, improving cortical excitability, increasing interneuronal connectivity, and brain remodeling, but the exact mechanism and standard treatment regimen are still inconclusive and controversial. Therefore, the review summarizes recent advances regarding the possible mechanisms, treatment options, precautions, and future trends of repetitive transcranial magnetic stimulation for the treatment of CPSP, to provide new ideas for better clinical work and scientific exploration.

## 1. Introduction

Stroke is characterized by high incidence, disability, and mortality rates.^[1]^ The prevalence of central poststroke pain (CPSP) following stroke ranges from 8% to 55%, significantly impairing patients’ mood, sleep, rehabilitation, and quality of life.^[2,3]^ Studies had demonstrated that even with the concurrent administration of high doses of various medications, the therapeutic outcomes for CPSP may remain unsatisfactory.^[4,5]^ In recent years, notable advancements had been made in treating CPSP through both invasive and noninvasive nerve stimulation techniques.^[6]^ However, invasive treatments carry substantial risks. Conversely, repetitive transcranial magnetic stimulation (rTMS) is relatively safe and has gained widespread clinical application.^[7]^ A meta-analysis of 6 studies (4 RCTs and 2 non-RCTs, involving 180 patients) concluded that rTMS significantly reduced CPSP compared to sham treatment.^[8]^ Another meta-analysis of 6 RCTs involving 288 patients also showed that rTMS significantly improved CPSP relative to placebo.^[9]^ Furthermore, Subgroup analysis revealed no statistically significant difference in pain relief lasting more than 6 months when comparing rTMS to conventional treatments. Currently, there is no consensus on the mechanisms or standardized treatment protocols for rTMS, which limits its broader adoption. Therefore, the review summarizes research progress on the possible mechanisms, treatment plans, and future trends of rTMS in the treatment of CPSP in recent years, to provide new ideas for clinical work and scientific exploration.

## 2. The pathogenesis of CPSP

As early as 1906, Dejerine and Roussy defined CPSP as a pain syndrome resulting from thalamic involvement following a stroke. Subsequent studies had shown that the spinal thalamic pathway could also lead to CPSP.^[10]^ In recent years, research had revealed that CPSP was a chronic neurological syndrome characterized by central nervous system (CNS) damage poststroke, leading to neurochemical changes that influence central sensitization, disinhibition, and alterations in both spinal and thalamic pathway.^[11]^ CPSP may result from CNS neurons responding to afferent impulses rather than being driven by ectopic CNS activity.^[12]^ To proved this hypothesis, Haroutounian et al injected lidocaine locally to block peripheral sensory input from painful limbs in 8 CPSP patients in a prospective study and found that 50% of patients experienced pain relief within 30 minutes.^[12]^ This finding suggested that pain may not occur spontaneously in the CNS itself as previously assumed, but rather may be secondary to the CNS’s misinterpretation of surrounding sensory signals. CPSP was the result of multifactorial dysfunction in interconnected neural networks, and its exact mechanism remained for further investigation.

## 3. Clinical manifestations and characteristics of CPSP

Poststroke pain can manifest in various forms, including CPSP, painful spasms, hemiplegia, tension headaches, and musculoskeletal pain.^[2,10,13]^ CPSP typically develops within 1 to 2 months after a stroke, although some cases may not manifest until 1 to 6 years later.^[11]^ CPSP has no characteristic manifestations, which is persistent or intermittent, and almost all patients have temperature and pain abnormalities. Nearly all patients experience temperature and pain abnormalities, often described as combinations of burning, cold, knife-like, compressive, or needle-like sensations. Pain distribution can range from a single point to encompassing the entire trunk or face.^[4]^ A retrospective study of 299 consecutive stroke patients reported that during a 6-month follow-up, 45.8% of patients experienced new-onset pain: headache (13.1%), shoulder pain (16.4%), other joint pain (11.7%), other pain (20.0%), light or heat-induced pain (8.0%), and 36.5% of patients had more than one type of pain.^[3]^ The presence of CPSP hinders the recovery of patients’ daily activities and can lead to emotional disorders, significantly impacting their quality of life.^[14]^ Due to the diversity of CPSP symptoms, it is challenging to distinguish it from other chronic pain diseases. In addition, cognitive impairment caused by stroke makes diagnosis more difficult.

## 4. Mechanism of rTMS in treating CPSP

**Figure 1.**
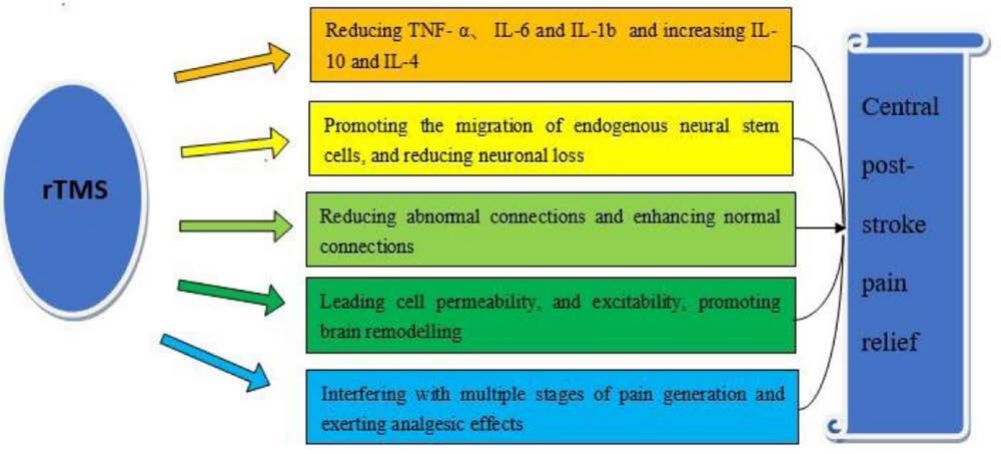
The mechanism of rTMS in the treatment of CPSP. rTMS = repetitive transcranial magnetic stimulation.

Transcranial magnetic stimulation utilizes electromagnetic coils to generate a magnetic field, which generates brief electromagnetic pulses that can easily and painlessly pass through the skull and enter the brain, inducing cortical excitatory changes through synapses at the stimulation site and a distance.^[15]^ Although widely used in clinical practice, the exact mechanism remains unclear. Current studies suggest several potential mechanisms (Fig. 1).

### 4.1. Regulating neuroinflammation

Immune cells can produce pro-nociceptive or antinociceptive mediators that regulate pain in various ways. Pro-inflammatory cytokines such as tumor necrosis factor (TNF)-α, interleukin (IL)-6, and IL-1B are mediators of inflammation and neuropathic pain, and anti-inflammatory cytokines such as IL-10 and IL-4 can regulate the inflammatory process, limit tissue damage, and restore homeostasis.^[16]^ These cytokines can regulate inhibitory and excitatory synaptic transmission, ultimately improving the transmission of pain signals to the brain. rTMS plays an important role in neuroinflammation by increasing the expression of anti-inflammatory cytokines and reducing the expression of pro-inflammatory cytokines.^[17,18]^

Stroke can induce nerve damage or vasodilation, resulting in the production of pro-inflammatory cytokines such as histamine, nerve growth factor, IL-10, and TNF in both the peripheral nervous system and CNS, thereby contributing to neuropathic pain.^[19]^ rTMS had been shown to reduce microglia activation and increase IL-10 levels in the cortex, alleviating neural abnormalities in a rat model of nerve injury.^[18]^ Other animal studies had demonstrated that rTMS (5–10 Hz) decreases the polarization of neurotoxic astrocytes and promotes neuronal regeneration in stroke rats by reducing IL-10 expression.^[20]^ In addition, rTMS also reduced TNF and IL-1β. By reducing the number of activated microglia in the cerebral cortex and promoting the secretion of calcitonin gene-related peptides, calcitonin gene-related peptides can promote rTMS to reduce infarct area and increase TNF and IL-1β.^[21]^

### 4.2. Promoting neuronal cell proliferation

Endogenous neural stem cells often migrate to the damaged area and differentiate into astrocytes after ischemic stroke, forming glial scars instead of neurons.^[22]^ Recent research indicated that rTMS increases the expression of stromal cell-derived factor-1α and chemokine receptor-4 in the ipsilateral primary motor cortex (M1), promoting the migration of endogenous neural stem cells and reducing neuronal loss in cortical tissue surrounding the infarction in ischemic stroke rats.^[23]^ A previous study also showed that rTMS acts on the ipsilateral hemisphere and human neural stem cells, and had a beneficial effect on functional recovery by increasing the expression of brain-derived neurotrophic factor after neurogenesis and ischemic stroke. Low-frequency transcranial magnetic stimulation and intermittent transcranial magnetic stimulation could promoted the conversion of pluripotent stem cells to mature neurons.^[24]^ In addition, high-frequency magnetic stimulation can enhanced the transcription of vesicular glutamate transporter-2 in neurons, which was associated with the neurogenic effects of transcranial magnetic stimulation.^[20]^

The impact of repetitive rTMS on microglia remains underexplored. Microglia, as resident immune cells of CNS, remain quiescent when not activated but possess migratory and phagocytic capabilities.^[25]^ Upon activation, microglia differentiate into pro-inflammatory or anti-inflammatory phenotypes, monitoring the CNS microenvironment and promptly eliminating necrotic neurons to maintain homeostasis.^[25]^ After the application of low-frequency (LF)-rTMS (1 Hz), the number of states of microglia in the motor cortex of healthy rats did not change. However, high-frequency (HF)-rTMS significantly increased the number of activated microglia in mongolian gerbils with cerebral ischemia, promoted neurogenesis, and improved stroke recovery.^[26]^ Furthermore, studies had shown that neural stem cells cultured in rTMS-treated microglial conditioned medium exhibit reduced apoptosis and enhanced neuronal differentiation.^[27]^

### 4.3. Adjusting the functional connections of the brain

CPSP is often linked to strokes affecting the somatosensory pathway, including structures such as the ventral posterolateral nucleus, anterior occipital nucleus, and lateral medullary nucleus. It may also involve changes in the medial emotional pathway, including the amygdala, anterior cingulate cortex, and insular cortex.^[28]^ Evidence from animal studies suggested that CPSP reduced the functional connection between ventral posterolateral nucleus and the somatosensory cortex (responsible for perceiving pain location, intensity, and duration), and increased the functional connection between the medial nucleus and amygdala (responsible for attention, cognition, and pain perception). rTMS alleviated and eliminated pain by reducing abnormal connections and enhancing normal connections.^[29,30]^ To verify the functional recombination hypothesis of CPSP development and the rTMS treatment mechanism, Yadono et al conducted a longitudinal study on 2 male model monkeys with unilateral thalamic ventrolateral nucleus hemorrhage-induced CPSP, tracking structural and functional changes in their brains.^[30]^ Applying rTMS to ipsilateral M1 could alleviated pain induced in model monkeys. Fibre bundle imaging analysis showed a decrease in structural connectivity within the ipsilateral thalamocortical tract, which was unaffected by rTMS. In addition, resting-state functional magnetic resonance imaging (fMRI) found abnormal enhancement of functional connectivity between the ipsilateral thalamus dorsomedial nucleus and amygdala, potentially contributing to CPSP. At the same time, rTMS can normalize enhanced connection, which may be the underlying mechanism of rTMS in treating CPSP.^[31,32]^

### 4.4. Regulating brain remodeling

rTMS acts on neuronal synapses, depolarizing axons and transmitting them downwards, leading to changes in the neuronal body, cell permeability, and excitability, promoting brain remodeling.^[7]^ The expression of BDNF increases in stroke patients with neuropathic pain, indicating its involvement in pain recovery after stroke.^[33]^ Moreover, various experimental research results emphasized that the reduction of GABAergic neurotransmission in CNS was the main cause of chronic neuropathic pain.^[34]^ rTMS optimizes neurotransmitter levels, increasing GABA concentration, promoting endogenous opioid release, and enhancing BDNF secretion, thereby improving pain management.^[35]^ Zhao et al applied 10 HZ rTMS to 40 patients with acute CPSP, and after 3 weeks of treatment, serum BDNF significantly increased and the pain was effectively relieved.^[36]^ Other studies also showed that rTMS (20 Hz) regulated the mRNA expression levels of neurotransmitter-related genes, including GABAergic, glutamate ergic, and glycine ergic neurotransmitter systems in the cerebellum and brainstem, achieving synaptic plasticity.^[26]^

High-frequency repetitive transcranial magnetic stimulation (HF-rTMS) increases intracortical inhibition and intracortical facilitation, which may contribute to the alleviation of CPSP.^[37]^ Lefaucheur et al conducted a study on 22 patients with neuropathic pain, demonstrating that HF-rTMS targeting the M1 not only reduced pain but also increased cortical inhibition. However, whether the conclusion was also applicable to patients with CPSP remains to be further verified.^[38]^ They speculated that rTMS regulated the balance between inhibitory neurotransmitters and excitatory glutamate neurotransmitters in the cerebral cortex, achieving analgesic effects.^[38]^

### 4.5. Reduce central sensitization

Central sensitization refers to an abnormal increase in excitability or synaptic transmission of pain-related neurons in CNS, including an increase in neuronal spontaneous discharge activity, expansion of sensory domains, and a decrease in the threshold for external stimuli, thereby amplifying the transmission of pain signals.^[39]^ rTMS exerts its analgesic effects by interfering with multiple stages of pain processing through advanced regulation of CNS, acting on the cerebral cortex and adjacent subcortical structures.^[40]^ Yang study including thirty-two male Sprague-Dawley rats showed that HF-rTMS reduced central sensitization and alleviated neuropathic pain by downregulating the overexpression of neuronal nitric oxide synthase in the ipsilateral dorsal root ganglia and inhibiting the activity and proliferation of astrocytes in the ipsilateral L4 to L6 spinal dorsal horn.^[41]^

## 5. Clinical application of rTMS in the treatment of CPSP

Currently, there is no standardized treatment protocol for rTMS in CPSP, as different parameters such as frequency, site, and duration of stimulation yield varying analgesic outcomes.^[42]^ The commonly used clinical modes include HF-rTMS, LF-rTMS, and θ burst stimulation. The neuropathic pain disorder has obvious pain intermittency, and treatment at different stages may have different effects. Compared with stimulation during the period of rising pain, rTMS administered at the time of pain initialization can produce better analgesic effects.^[43]^ Especially for patients with chronic pain, choosing the best treatment time to minimize pain is more meaningful and convenient.

### 5.1. rTMS treatment plans for CPSP

Higher frequencies (≥5 Hz) can increase cortical excitability, while lower frequencies (≤1 Hz) can reduce cortical excitability. Different stimulation frequencies are selected based on the stimulation site and the patient’s pain status.^[44]^ In multiple studies on the analgesic effect of rTMS on CPSP, it had been found that HF-rTMS (5–20 Hz) was more effective in alleviating CPSP than LF-rTMS. The reduction in visual analogue scale (VAS) and digital scale scores was significantly greater with HF-rTMS than with LF-rTMS, and multiple, longer interventions produced better analgesic outcomes compared to single, short-term interventions. Therefore, HF-rTMS was often used in clinical practice to treat CPSP.^[45–47]^

#### 5.1.1. HF-rTMS stimulation site

Due to the various mechanisms involved in treating CPSP with rTMS, the treatment plans developed as a result have not been unified. In a prospective, randomized, double-blind trial, patients were divided into 3 groups based on target locations, such as M1, secondary motor cortex (S2), and placebo group. Each group received 10 courses of rTMS treatment, with 5050 pulses per course and a frequency of 10 Hz.^[48]^ The results showed that all patients experienced short-term pain relief (17–20% relief), but there was no statistical difference between the groups, indicating a strong placebo effect in pain relief. Additionally, only patients with S2 as the target area had significant long-term pain relief (15% pain relief). Zhao et al randomly divided 40 patients into rTMS (10 Hz, 2000 stimuli) (n = 20) or sham intervention (n = 20) for 3 weeks, with the stimulation site being the motor cortex responsible for the upper limb and hand regions. The results confirmed that intervention in these areas effectively alleviated CPSP.^[36]^ In the other 2 prospective randomized controlled trials, the stimulation sites of the dorsolateral prefrontal cortex and anterior cingulate cortex did not produce effective analgesic effects compared to the stimulation of the motor cortex, and the specific reasons are not yet clear.^[49,50]^

In recent years, many scholars had used fMRI and TMS to identify the brain regions affected by stroke and the optimal regions for rTMS stimulation, which also evaluated the therapeutic effect of rTMS on brain function and neural connectivity in stroke patients.^[31]^ Imaging technology develops personalized rTMS treatment plans for every patient based on damaged areas of the brain. For example, fMRI can identify brain regions that were still active after a stroke, and then provide rehabilitation treatment for these regions.^[32]^ However, Due to significant differences in examination equipment and the experience of imaging physicians in hospitals, it is not yet widely applicable.

#### 5.1.2. HF-rTMS frequency, number, and interval time

In a randomized, double-blind study, Mori et al established 4 groups based on stimulation conditions: 5 Hz, 500 pulses per time, 10 Hz, 500 pulses per time, 10 Hz, 2000 pulses per time, and sham stimulation.^[51]^ The analgesic effect was evaluated using the VAS and the Simplified McGill Pain Questionnaire 2. The results showed that the 10 Hz rTMS group with 2000 pulses was more effective in alleviating neuropathic pain than the sham stimulation group and low-dose stimulation group. Similarly, in another study, patients were treated with 10 Hz rTMS with 1000 pulses per day for 10 consecutive days, resulting in a significant decrease in VAS, which decreased from 7 to 5.6 in the second week and 3.9 in the 8th week.^[52]^ Some scholars also believed that 4 consecutive rTMS treatments at a frequency of 20 Hz in the M1 region every 3 weeks alleviate refractory CNS pain.^[53]^ On the contrary, another study found that continuous rTMS of 5 courses per day was ineffective in relieving various central pain patients.^[54]^ More large-scale, multicenter, prospective, and randomized controlled trials are needed to clarify the specific standardization plan.

### 5.2. θ burst stimulation therapy for CPSP

θ burst stimulation is a new mode of prolonging the effectiveness of rTMS. Intermittent θ burst stimulation (iTBS) has excitability while continuous θ burst stimulation has inhibitory properties, and the intensity of iTBS stimulation is lower than the threshold of motor evoked potential, making it less likely to cause epilepsy.^[55]^ iTBS causes long-term synaptic changes and may be more effective in long-term pain relief than rTMS.^[56]^ In a study conducted in South Korea, 30 patients were randomly assigned to the iTBS group and control group. Each patient underwent 5 rounds of iTBS treatment, and digital scales and neurological pain symptom scales were scored before and after treatment. The results indicated significant reductions in both scale scores for the iTBS group, whereas no significant changes were observed in the control group.^[57]^

## 6. Contraindications to rTMS treatment for CPSP

Magnetic fields may cause malfunction or malfunction of intracranial implantation equipment, so the absolute contraindication for rTMS is intracranial metal hardware, such as brain stimulators, electrodes, epidural cortical stimulators, aneurysm clips, coils, stents, cochlear implants, pacemakers, implantable cardioverter defibrillators, and spinal cord stimulators.^[58]^ However, some studies also showed that as long as the internal pulse generator was not close to the rTMS coil, rTMS was relatively safe for implantable electronic devices.^[59]^ Further large-scale research with long-term follow-up is necessary to verify these findings. Although there is no clear evidence of adverse effects on fetuses, most scholars do not recommend using rTMS during pregnancy.^[60]^

## 7. Adverse reactions

Common adverse reactions include scalp discomfort, dizziness, headache, nausea, tinnitus, and hearing loss, often ranging from mild to moderate in severity, occasionally encountering serious events such as seizures and syncope.^[61]^ According to statistics, headache is the most common adverse event (9.7%), followed by scalp discomfort (9.3%) and nausea (5%).^[61,62]^

The clicking sound during rTMS can significantly increase the auditory threshold and interfere with the auditory function after stimulation. After a single rTMS treatment of 20 minutes, the increase in the auditory threshold generally does not exceed 1 hour.^[63]^ The probability of rTMS-inducing seizures is extremely low and usually self-limiting.^[64]^ The estimated risk of seizures during treatment in patients receiving HF-rTMS, LF-rTMS, iTBS, and continuous θ burst stimulation is 0.05%, 0.03%, 0.06%, and 0%, respectively.^[61]^ There are no reports of seizures when rTMS is administered to individuals without risk of seizures, such as brain tumors, severe head injuries, concussions, neurological disorders, or drugs that lower seizure thresholds.^[64]^

## 8. Conclusion and prospects

rTMS has a good analgesic effect on CPSP, potentially attributable to its multifaceted mechanisms, including modulation of immune responses, promotion of neurogenesis, enhancement of cortical excitability, augmentation of neural connectivity, and facilitation of brain remodeling. Most research results indicated that iTBS, HF-rTMS, and multi-course rTMS had better therapeutic effects than LF-rTMS and single-course rTMS (Table 1). However, the majority of current studies had been constrained by small sample sizes, thereby limiting the robustness and reliability of their conclusions. To allow translation into health care, it requires larger studies or pooled data. Brain stimulation consortia such as ENIGMA may be a crucial step in this resource-intensive process. Future research will aim to elucidate the precise mechanisms underlying rTMS treatment for CPSP, with the goal of achieving targeted rehabilitation based on pathophysiological insights.

**Table 1:** The clinical studies on the application of rTMS in treating CPSP.

Year and authors	Journal	Sample size	Diseases	Main findings	Study type
Lefaucheur et al^[38]^	Neurology	22 patients	Chronic neuropathic pain	10 Hz rTMS significantly improved pain and enhanced intracortical inhibition	RCT
Kim et al^[32]^	Stroke	20 patients	Chronic stroke	rTMS enhanced corticomotor excitability and promoted motor recovery	Prospective
Galhardoni et al^[49]^	Neurology	54 patients	Central neuropathic pain	Insular stimulation not superior to M1; supports M1 as preferred target	RCT
Hosomi et al^[54]^	Pain	36 patients	Neuropathic pain	Daily sessions (5/week) more effective than weekly sessions for pain reduction	RCT
Attal et al^[50]^	Brain	161 patients	Refractory neuropathic pain	M1-targeted rTMS significantly reduced pain vs sham (*P* < .01)	Multicenter RCT
Zhao et al^[36]^	Pain Ther	40 patients	Acute CPSP	10Hz rTMS (2000 pulses) ↓ VAS scores (*P* < .001) and ↑ serum BDNF	RCT
Ojala et al^[48]^	Neuromodulation	20 patients	CPSP	M1 and S2 targets provided short-term relief; S2 showed superior long-term effects	RCT (pilot)
Mori et al^[51]^	Neuromodulation	48 patients	Neuropathic pain	10 Hz/2000 pulses optimal for analgesia (VAS↓ >30%)	RCT
Lin et al^[52]^	J Neurol	382 patients	Stroke with neglect	rTMS improved neglect by modulating neural networks (meta-analysis of 12 studies)	Meta-analysis
Gurdiel-Alvarez et al^[8]^	Front Neurosci	180 patients	CPSP	rTMS significantly reduced CPSP pain (SMD = ‐0.89, 95% CI [‐1.21,‐0.57])	Meta-analysis
Liu et al^[9]^	Front Neurosci	288 patients	CPSP	HF-rTMS (>5Hz) superior to LF-rTMS for pain relief (*P* < .01)	Meta-analysis
Tamasauskas et al^[6]^	J Pain	–	CPSP	rTMS recommended as first-line non-pharmacological therapy (Grade A)	Meta-analysis
Lou et al^[1]^	Stroke Vasc Neurol	–	Stroke patients	rTMS recommended for CPSP (Class IIa) in clinical guidelines	Guideline

CPSP = central poststroke pain, LF = low-frequency, rTMS = repetitive transcranial magnetic stimulation, VAS = visual analogue scale.

## Author contributions

**Resources:** Tao Liu, Feiye Chen.

**Software:** Tao Liu.

**Writing – original draft:** Nannan Yang.

**Writing – review & editing:** Nannan Yang, Deheng Cui.
